# Evaluating the benefits of adjuvant chemotherapy in patients with pancreatic cancer undergoing radical pancreatectomy after neoadjuvant therapy—a systematic review and meta-analysis

**DOI:** 10.3389/fonc.2024.1429386

**Published:** 2024-10-17

**Authors:** Jiahao Wu, Yike Zhang, Haodong Wang, Wenyi Guo, Chengqing Li, Yichen Yu, Han Liu, Feng Li, Lei Wang, Jianwei Xu

**Affiliations:** Department of Pancreatic Surgery, General Surgery, Qilu Hospital of Shandong University, Jinan, China

**Keywords:** adjuvant chemotherapy, neoadjuvant therapy, pancreatic ductal adenocarcinoma, overall survival, disease-free survival, TNM-staging

## Abstract

**Background:**

More and more patients with pancreatic cancer (PC) received neoadjuvant therapy (NAT) and then underwent radical pancreatectomy. However, the benefit of adjuvant chemotherapy (AC) for these patients is still controversial. This study is designed to determine the benefits of postoperative AC for patients with PC undergoing NAT and radical resection.

**Methods:**

We conducted a comprehensive search of the PubMed, Embase, Web of Science, and Cochrane Library databases, covering the period from their inception until 10 September 2023. Our analysis focused on the assessment of overall survival (OS) and recurrence-free survival (RFS) through meta-analysis. The fixed-effects model and the random-effects model were used to process the data. Hazard ratios (HRs) and 95% confidence intervals (95% CIs) were employed to determine the necessary of administering AC for patients with PC who have undergone NAT and radical resection. We retrieved 3,063 search results, of which 3,048 were excluded because of duplication or after applying our inclusion and exclusion criteria.

**Results:**

A total of 15 studies with 21,113 patients (7,794 patients in the AC group and 13,319 in the non-AC group) were included, all of which reported OS, and three studies reported disease-free survival (DFS)/tumor-specific survival (CSS)/RFS. The final results showed that AC significantly improved OS and DFS/CSS/RFS in patients with PC who underwent pancreatectomy after NAT [OS: HR = 0.80, 95% CI (0.75∼0.86), P < 0.00001, I^2^ = 48%; DFS/CSS/RFS: HR = 0.53, 95% CI (0.41~0.69), P < 0.00001, I^2^ = 0%]. Furthermore, we performed subgroup analyses and demonstrated that AC provided a significant survival benefit for patients with PC after NAT and resection regardless of the tumor size [<2-cm subgroup: HR = 0.72, 95% CI (0.5∼0.94), P = 0.01; ≥2-cm subgroup: HR = 0.79, 95% CI (0.65∼0.96), P = 0.02] and the margin status [R0 subgroup: HR = 0.83, 95% CI (0.77∼0.88), P < 0.00001; R2 subgroup: HR = 0.75, 95% CI (0.61∼0.92), P = 0.007]. AC also benefited the patients with a stage N0 [HR = 0.79, 95% CI (0.74~0.84), P < 0.00001], N1 [HR = 0.78, 95% CI (0.72∼0.85), P < 0.00001], or poorly/undifferentiated tumor [HR = 0.76, 95% CI (0.66∼0.87), P < 0.0001] in survival but not in patients with a stage N2 [HR = 0.69, 95% CI (0.43∼1.09), P = 0.11] or well/moderately differentiated tumor [HR = 0.97, 95% CI (0.66∼1.42), P = 0.87].

**Conclusions:**

Although AC showed survival benefit for patients with PC undergoing radical pancreatectomy after NAT, we still need to consider the lymph node stage and the degree of differentiation of the tumor when we gave AC to a patient. High-quality prospective randomized controlled studies are required to well disclose the value of AC in patients with PC undergoing radical pancreatectomy after NAT.

**Systematic review registration:**

https://www.crd.york.ac.uk/prospero/ PROSPERO, identifier CRD42023461365.

## 1 Introduction

Pancreatic cancer (PC) is one of the most aggressive and lethal malignancies with a 5-year overall survival (OS) of less than 10% (1). Given the high mortality and increasing incidence every year, PC is projected to become the second leading cause of cancer-related death by 2030 (2). Currently, surgical resection is still the only radical treatment for PC. However, the effect of operation is not good, the 5-year OS is as low as 20%, whereas about 75% of patients will experience tumor recurrence within 2 years (3–5). Systemic adjuvant chemotherapy (AC) plays a crucial role in treatment of patients underwent radical resection in considering that PC is a systemic disease (6). Disappointingly, the improvement of systemic AC on the survival of patents after radical resection is still limited (7).

Neoadjuvant therapy (NAT) has been become the important treatment for patients with localized PC (8), which downstages the primary tumor, increases the feasibility of R0 resection, eliminates micrometastasis, and identifies aggressive tumors to avoid futile surgery (9, 10). Moreover, nearly half of the patients are unable to receive AC due to surgical complications after operation (11), and preoperative chemoradiotherapy successfully overcomes this situation. The prolonged disease-free survival (DFS) and OS also confirms the advantages of NAT in patients with high risk resectable and borderline resectable PC (12, 13).

However, the necessity and benefits of AC in patients with PC underwent pancreatectomy after NAT remains controversial. Sugawara et al. found that patients with PC received AC after NAT, and resection had significantly better survival benefits than those did not receive AC (14). In contrast, van Roessel’s team indicated that additional postoperative therapy may not provide an additional survival benefit, except for patients with pathologically confirmed lymph node-positive PC (15).

To well disclose the value of AC in patients with PC underwent pancreatectomy after NAT, we conduct a systematic review and meta-analysis, the effects of AC on survival and the potential benefit subgroups will be reported.

## 2 Materials and methods

### 2.1 Literature search strategy and selection criteria

The PubMed, Embase, Web of Science, and Cochrane Library databases from inception through 10 September 2023 were searched for literature published in English comparing the need for postoperative AC for patients with PC after NAT. We used the following terms: (pancreatic neoplasm OR pancreas cancer) AND (neoadjuvant therapy OR neoadjuvant Chemotherapy OR neoadjuvant chemoradiotherapy) AND (adjuvant chemotherapy OR adjuvant drug therapy). The detailed search strategy is summarized in **Supplementary Table 1**. In addition, all eligible studies were manually scrutinized. Two investigators independently evaluated the included studies. Any discrepancies in the literature search were settled by a consensual process.

Studies that meet the following criteria could be included: (1) the study design was a randomized controlled clinical trial or prospective/retrospective cohort study; (2) the study subjects were all patients with PC who undergo surgical resection after NAT (R2 resections < 2% per study; AC cycle > 3 months); (3) the studies had sufficient data to be analyzed including clinical characteristics and prognostic indexes such as OS, DFS, recurrence-free survival (RFS) and tumor-specific survival (CSS); (4) the study was published in English. Exclusion criteria are as follows: (1) meta-analysis, review, case report, comment, letter, conference abstract, and ongoing studies; (2) animal experiment and study not related to the subject matter of the article; and (3) studies that did not provide enough information to be included. For republished studies, only the most recent literature and relevant data were collected. Study design [Participants/Patients Intervention Control/Comparison Outcome Study design (PICOS)] components are detailed in **Supplementary Table 2**.

This study was conducted in accordance with the criteria established by the Preferred Reporting Items for Systematic Reviews and Meta-Analyses (PRISMA) statement (supplementary PRISMA Checklist). It was registered in the PROSPERO (CRD42023461365) prospectively (16).

### 2.2 Data analysis

Two researchers (JHW and YKZ) independently extracted data and performed a literature quality assessment. Any disagreements were resolved by consensus through discussion with a third investigator. We extracted baseline characteristics from the included literature, including first author, study period, study country, year of publication, sample size, clinical characteristics, and clinical outcomes. The study selected OS and DFS/CSS/RFS as endpoints for this meta-analysis. The quality of the included studies was assessed using the Newcastle–Ottawa Scale (NOS) (17), where seven to nine points were rated as high-quality studies.

The hazard ratios (HRs) and 95% confidence intervals (95% CIs) were used to estimate the correlation between the administration of AC or not and the patient’s prognosis in patients with Pancreatic Ductal Adenocarcinoma (PDAC) undergoing surgical resection after NAT. In most studies, data such as HRs and 95% CIs could be collected directly. However, we used Tierney’s method to derive estimates from survival curves for all studies without relevant prognostic indicators (18). The heterogeneity among the included studies was assessed by using the chi-squared (χ^2^ ) test (Cochran’s *Q*) and inconsistency index (I^2^) (19). P-values < 0.05 or I^2^ > 50% indicated significant heterogeneity, in which case it was analyzed by using a random-effects model. Conversely, fixed-effects models were used when heterogeneity was small. P < 0.05 was considered statistically significant. Sensitivity analyses, funnel plots, and subgroup analyses were used to detect sources of heterogeneity. The subgroup analyzed factors included study style, the American Joint Committee on Cancer (AJCC) eighth N staging, tumor size (<2 cm and ≥2 cm), margin status, and histological grade. The results of the subgroup analysis are presented in **Table 1**. Begg’s funnel plot test and Egger’s test were used to test for publication bias in these studies (20). RevMan 5.3 (Cochrane Collaboration) and Stata 16.0 software (College Station) were used for this meta-analysis.

**Table 1:** Subgroup meta-analysis of prognostic role of AC for OS in PC patients after NAT and radical pancreatectomy.

Factor	No. of study	No. of study		HR (95%CI)	P-value	Heterogeneity	
		AC	Non-AC			I² (%)	P-value
Study Style							
Retrospective	12	12	12	0.82	<0.00001	41%	0.2
Prospective	3	3	3	0.64	0.004	38%	<0.06
AJCC 8th N staging							
N0	9	9	9	0.79	<0.00001	0%	0.65
N1	6	6	6	0.78	<0.00001	32%	0.19
N2	4	4	4	0.69	0.11	75%	0.07
Tumor size							
<2cm	3	3	3	0.72	0.01	0%	0.48
<2cm	3	3	3	0.79	0.02	59%	0.09
Margin status							
R0	4	4	4	0.83	<0.00001	16%	0.31
R1	4	4	4	0.75	0.007	51%	0.11
Histological grade							
Low-grade group	3	3	3	0.97	0.87	0%	0.8
High-grade group	3	3	3	0.76	<0.00001	15%	<0.31

## 3 Results

**Figure 1:**
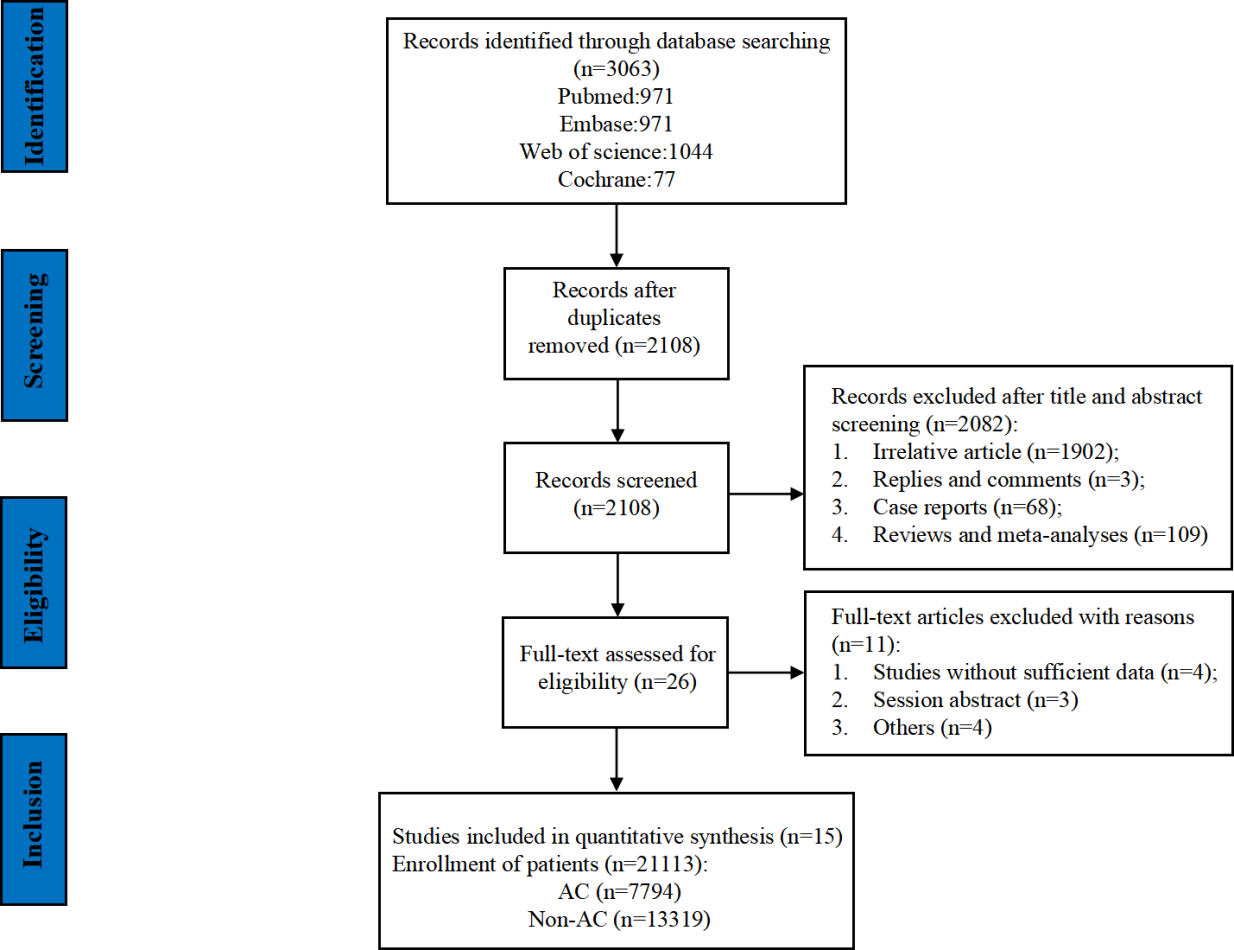
Flowchart of the systematic search and selection process.

The flowchart of the literature retrieval and screening process is presented in **Figure 1**. The systematic initial search yielded 3,063 relevant literatures, of which 955 were excluded due to duplication. Subsequently, 2,082 articles were elected by title and abstract, and 26 remaining articles were subjected to full text examination. Finally, a total of 15 articles that met the inclusion criteria were included in our meta-analysis, and a pooled analysis of 21,113 patients was conducted (7,794 patients in the AC group and 13,319 patients in the non-AC group). Three of these studies are retrospective ones using prospectively maintained databases (21–23), and the other 12 were retrospective cohort studies (14, 15, 24–33). It was worth noting that the study by Bolm et al. analyzed two sets of data separately. The included studies were published between 2017 and 2023 and conducted in five countries: 10 in the United States (14, 15, 21–24, 26, 28, 30, 31), 2 in China (32, 33), and 1 each in South Korea (27), Italy (29), and The Netherlands (25). **Table 2** provides a summary of the main characteristics and NOS scores of the included studies. The median NOS score was 7 (ranging from 6 to 9) (34), and 10 studies were assessed as high-quality. **Supplementary Table 3** presents the NOS assessment details for all included studies.

**Table 2:** The characteristics of the included studies.

Study	Year	Country	Study style	Sample size	Patient		Years of enrolled patients	Median OS		Outcome	Follow-up times	NOS score
					Non-AC	AC		Non-AC	AC			
Olecki et al. (30)	2021	USA	Retrospective	3,897	2,405	1,492	2006–2015	23.22	26.04	OS	NA	7
Kamarajah et al. (26)	2021	USA	Retrospective	4,122	2,061	2,061	2004–2016	24.9	29.4	OS	NA	8
Ma et al. (28)	2019	USA	Retrospective	1,737	1,247	490	2004–2015	NA	NA	OS	Median time, 36 months	8
Sugawara et al. (14)	2023	CA	Retrospective	888	444	444	2010–2018	21.2	26.6	OS	Median time, 21.3 months	9
Bolm et al. (24)	2022	USA	Retrospective	202	100	102	2000–2018	17.8	21.3	OS	NA	6
Bolm* et al. (24)	2022	USA	Retrospective	4,749	4,172	577	2004–2018	26.4	35.4	OS	NA	6
Zhang et al. (33)	2022	USA	Retrospective	764	561	203	2011–2017	28.9	30.1	OS	Median time, 35.5 months	7
van Roessel et al. (15)	2020	EU	Retrospective	520	177	343	2012–2018	29	29	OS	Median time, 38 months	7
Hammad et al. (22)	2023	USA	Prospective	413	199	214	2010–2019	NA	NA	OS/PFS	NA	6
Barnes et al. (21)	2017	USA	Prospective	234	96	138	2009–2016	39	42.3	OS/PFS	Median time, 25.2 months	7
de Geus et al. (25)	2018	USA	Retrospective	1,357	833	524	2006–2013	27.1	27.5	OS	NA	6
Maggino et al. (29)	2023	ITA	Retrospective	373	123	250	2013–2017	35	36	OS	NA	6
Perri et al. (31)	2020	USA	Retrospective	122	61	61	2010–2017	17	42	OS/PFS	NA	8
Ivey et al. (23)	2022	USA	Prospective	427	186	241	2015–2019	20.4	28.7	OS/PFS	NA	6
Pu et al. (32)	2023	CA	Retrospective	1,194	597	597	2006–2019	25	30	OS/CSS	NA	8
Lee et al. (27)	2023	KR	Retrospective	114	57	57	2017–2020	23.5	31.5	OS/DFS	Median time, 26.6 months	9

NOS, the Newcastle-Ottawa Scale.

NA, Not Applicable.

*It was worth noting that Bolm et al. study analyzed two sets of data separately.

**Figure 2:**
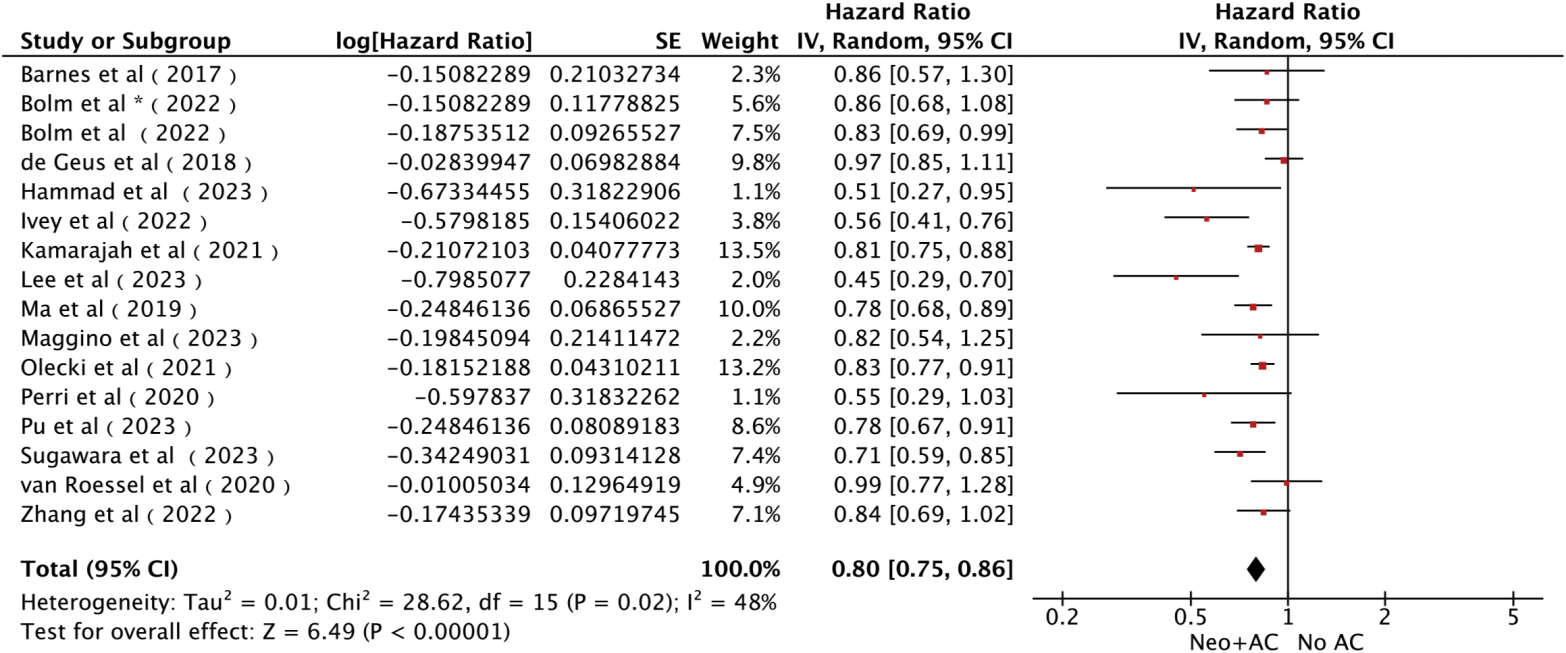
Forest plots of OS in patients with PC who received AC after NAT and radical pancreatectomy. *It was worth noting that Bolm et al. study analyzed two sets of data separately.

**Figure 3:**
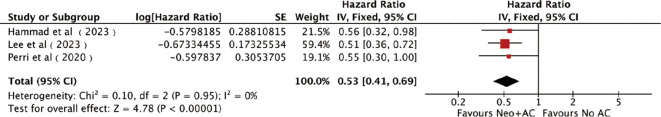
Forest plots of DFS/CSS/RFS in patients with PC who received AC after NAT and radical pancreatectomy.

All 15 studies (14, 15, 21–33) with a total of 21,113 patients reported the influence of AC on OS. The pooled results of all the cohort studies using a random-effects model showed that AC was associated with significantly longer OS [HR = 0.80; 95% CI (0.75~0.86), P < 0.00001; **Figure 2**], accompanied by slight heterogeneity (I^2^) = 48%, P = 0.02). Sensitivity analyses were performed to identify potential sources of heterogeneity, but no significant differences were found outside the limits of the 95% CI of the combined results. We also assessed the publication bias using funnel plots (**Supplementary Figure S2C**), Egger’s test, and Begg’s test and found no apparent publication bias for OS analysis (Egger’s test: P = 0.104, **Figure 3**; Begg’s test: P = 0.115, **Supplementary Figure S2A**).

Three studies (22, 27, 31), including 649 patients (332 patients in the AC group and 317 patients in the non-AC group), reported the DFS/CSS/RFS. The combined results obtained by the fixed-effects model showed a significant clinical benefit of AC on DFS/CSS/RFS in patients [HR = 0.53, 95% CI (0.41 ~ 0.69), P < 0.00001; **Figure 3**]. No heterogeneity was detected among the pooled results (I^2^) = 0%, P = 0.95). Sensitivity analyses also did not find differences in the pooled results beyond the limits of 95% CI. Funnel plots (**Supplementary Figure S2F**), Egger’s test, and Begg’s test did not find any obvious publication bias between AC and DFS/CSS/RFS (Egger’s test: P = 0.145, **Supplementary Figure S2F**; Begg’s test: P = 1.00, **Supplementary Figure S2D**).

Subgroup analyses about oncological factors was performed to determine the benefiting subpopulation, which was helpful to making a rational decision in application of AC for patients underwent radical resection after NAT. Additionally, all of the following subgroup analyses were analyzed using a random-effects model (**Table 2**), and publication bias of the included studies was assessed using Funnel plots, Egger’s test, and Begg’s test (**Supplementary Figure S3**).

**Figure 4:**
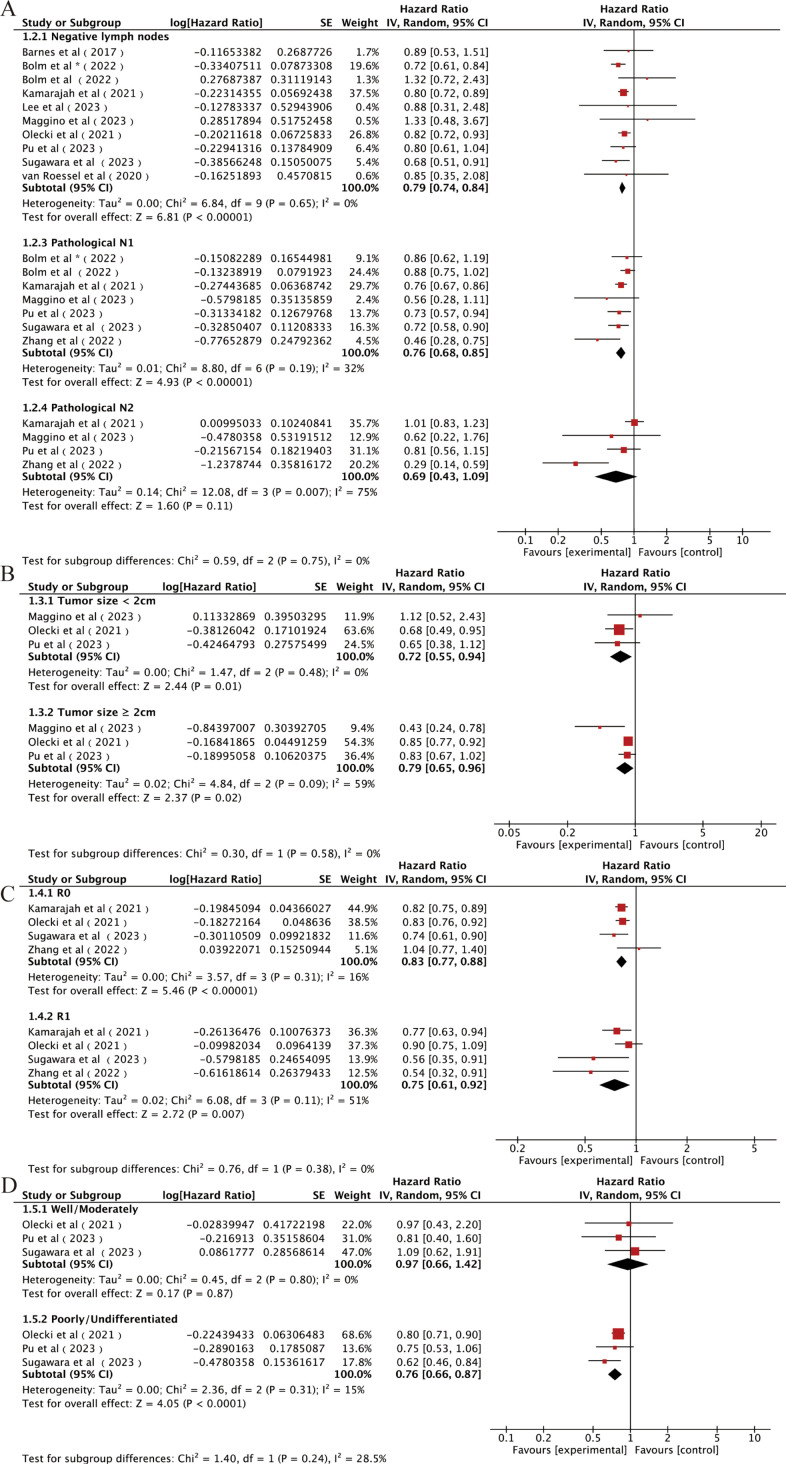
Forest plots of **(A)** N0, N1, and N2; **(B)** tumor size < 2 cm and tumor size > 2 cm; **(C)** R0 and R1; **(D)** well/moderate differentiation and poor/undifferentiation. *It was worth noting that Bolm et al. study analyzed two sets of data separately.

Firstly, we classified the included studies according to AJCC eighth N staging, and nine studies reported the effect of postoperative AC or OS in patients with a pathological N0 disease (14, 15, 21, 24, 26, 27, 29, 30, 32). The combined results using a random-effects model showed that patients with stage N0 could be significantly benefited from AC after NAT and surgery [HR = 0.79, 95% CI (0.74~0.84), P < 0.00001; **Figure 4A**]. Six studies reported the influence of postoperative AC on OS in patients with stage N1 (14, 24, 26, 29, 32, 33). The combined results also showed significant benefits of AC in these patients [HR = 0.78, 95% CI (0.72∼0.85), P < 0.00001; **Figure 4A**]. Four studies reported the effect of postoperative AC in patients with stage N2 (26, 29, 32, 33). Surprisingly, AC did not prolong OS in patients with an N2 tumor after NAT and surgery [HR = 0.69, 95% CI (0.43∼1.09), P = 0.11; **Figure 4A**].

Secondly, we analyzed the influence of tumor size on the effects of AC in patients underwent radical resection after NAT. Three studies were included (29, 30, 32), and the combined results indicated a significant improvement in OS associated with the use of AC regardless of the tumor size [<2-cm subgroup: HR = 0.72, 95% CI (0.55∼0.94), P = 0.01; ≥2-cm subgroup: HR = 0.79, 95% CI (0.65∼0.96), P = 0.02; **Figure 4B**].

Then, we analyzed the influence of the surgical margin status on the effects of AC in patients underwent radical resection after NAT. Four studies included (14, 26, 30, 33), and the combined results indicated that AC after NAT and resection increased OS compared with non-AC regardless of the margin status [R0 subgroup: HR = 0.83, 95% CI (0.77∼0.88), P < 0.00001; R2 subgroup: HR = 0.75, 95% CI (0.61∼0.92), P = 0.007; **Figure 4C**].

Finally, we analyzed the influence of tumor differentiation degree. Three studies were included, and the patients were classified to the low-grade group (well or moderate differentiation) and the high-grade group (poor or undifferentiation) according to the histological grade after completion of NAT. The combined results showed that the survival benefits of receiving additional AC after NAT were observed only in the high-grade group [HR = 0.76, 95% CI (0.66∼0.87), P < 0.0001; **Figure 4D**] (14, 30, 32) but not in the low-grade group [HR = 0.97, 95% CI (0.66∼1.42), P = 0.87; **Figure 4D**].

## 4 Discussion

To our knowledge, this study is the first one of systematic review and meta-analysis to assess the clinical implication of postoperative AC in patients with PC who underwent NAT and radical resection. The pooled analysis showed that AC was associated with a notably prolonged OS and DFS/CSS/RFS compared with non-AC. Subgroup analyses demonstrated that significant survival benefits of AC were observed regardless of the tumor size and resection margin status. However, the value of AC cannot be generalized in patients with different N staging and differentiation degree. We showed that AC benefited the patients with a stage N0, N1, or poorly/undifferentiated tumor in survival but not in patients with a stage N2 or well/moderately differentiated tumor. These findings were helpful to making a rational decision in selecting patients who underwent radical resection after NAT for further AC, which were also served as an important benchmark for future randomized controlled trials to well stratify patients.

PC has been emphasized as a systemic disease with a tendency to spread early (35, 36). Although surgery can offer a chance of cure for patients with (borderline) resectable PC, the presence of minimal residual and circulating tumor cells always results in early recurrence. It is worth noting that one of the most important roles of NAT, in addition to downstaging the primary tumor and increasing the rate of negative surgical margins, is the prevention of early postoperative metastasis in patients with PC (37). Furthermore, NAT has been shown to be efficient in enhancing systemic immunity and eradicating residual metastases in preclinical studies (38). Therefore, NAT can well overcome the limitations of the surgery-first approach, which has become a common practice in managing patients with borderline resectable PC and resectable tumors with high-risk factor and significantly prolongs the OS and Progression-free Survival (PFS) (39).

Noteworthily, there is evidence suggesting that the effects of trauma or immunosuppression in the host can cause metastasis of otherwise dormant tumor cells (40). As a trauma approach, preclinical evidence indicated that surgery could promote tumor metastatic mechanisms, potentially contributing to disease progression (41). AC could well remedy the shortcomings of surgery-only approach. However, whether the dormant tumor cells could be eliminated by NAT or the surgical-related trauma could arouse these cells was unknown. Whether AC could be replaced by NAT or AC could benefit the patients underwent radical resection after NAT was still unclear, especially considering the AC-related adverse effects and some postoperative patients unable to achieve the physical conditions required for AC, the value of AC in patients underwent radical resection after NAT was worth investigated.

Stereotypically, additional AC after NAT and surgery could provide an improvement in patients’ prognosis. Actually, the indications for this paradigm remain controversial. Several retrospective cohort studies about this issue have been published in recent years. Barnes et al. (21) reported a retrospective study that indicated AC after NAT and surgery did not improve the OS of the whole^234^. Similarly, van Roessel et al. (15) showed no significant difference in survival between patients with PC received additional AC and those without AC. On the contrary, the study conducted by Sugawara et al. (14) who analyzed the data-based National Cancer Database (NCDB) and the another retrospective study performed by Kamarajah et al. (26) indicated that AC after NAT and surgery was significantly associated with an improvement in survival. After pooling the publications, we showed that AC in patients with PC who underwent NAT and radical resection improve the OS compared with non-AC.

Even so, the favorable outcomes of AC still cannot be generalized for all patients, which were influenced by the metastasis status of the regional lymph node and the differentiation degree. It is worth noting that the value of AC in patients with PC with different N stages is still under debate. Sugawara et al. (14) showed that AC had a better survival benefit in patients with any N staging. Other some studies indicated an improved survival of additional postoperative AC in patients with regional lymph node metastasis (15, 21). However, Pu et al. (32) found that only patients with PC with N1 disease could significantly benefit from additional AC after NAT and surgery, rather than patients with N0 or N2 disease. In addition, Zhang et al. (33) reported that additional AC was not only unrelated to the survival of patients underwent NAT and surgery but even shortened the OS in patients with positive nodal disease. The current pooled results demonstrated that AC benefited patients with a tumor in the stage N0 and N1 in survival but not in patients with a stage N2 tumor. Similarly, the value of AC in patients with different degrees of differentiation, which improved the OS in patients with a poorly/undifferentiated tumor but not in patients with a well/moderately differentiated tumor. It is hard to explain the heterogeneity of the outcomes of AC in patients with different N stages or degrees of differentiation, further clinical study should focus on this phenomenon. This meta-analysis has several limitations. First, all studies included in our analysis were cohort studies, and there was a lack of large randomized controlled trials to enhance the level of evidence. Second, most of the studies were analyzed using the NCDB and SEER databases, which limited our ability to obtain detailed information on the specific regimens and treatment cycles of NAT and AC, as well as tumor characteristics of patients with PC, such as resectability assessment, vascular invasion, and other recurrence-related factors. Additionally, due to the constraints of retrospective data, information regarding tumor recurrence and its impact following AC, beyond survival data, remains inaccessible. Consequently, further prospective trials are necessary to examine the benefits of AC after NAT and surgery more thoroughly. This limitation hindered our ability to conduct a comprehensive analysis of NAT and AC regimens and individualized therapy. Finally, aside from OS, there was inconsistent reporting of outcomes across the 15 studies included in the meta-analysis. Thus, we were unable to utilize all the included articles for our analyses of other indicators.

## 5 Conclusions

In conclusion, in this meta-analysis of 15 cohort studies, although AC showed survival benefit for patients with PC undergoing radical pancreatectomy after NAT, we still need to consider the lymph node stage and the degree of differentiation of the tumor when we gave AC to a patient. High-quality prospective randomized controlled studies are required to well disclose the value of AC in patients with PC undergoing radical pancreatectomy after NAT.

## Data Availability

The original contributions presented in the study are included in the article/**Supplementary Material**. Further inquiries can be directed to the corresponding authors.
