# Supplementary material for: Evaluating the benefits of adjuvant chemotherapy in patients with pancreatic cancer undergoing radical pancreatectomy after neoadjuvant therapy—a systematic review and meta-analysis
Source: Front Oncol. 2024 Oct 17;14:1429386. doi: 10.3389/fonc.2024.1429386 (PMC11524795; doi:10.3389/fonc.2024.1429386)
Supplement: Supplementary file 1 [file DataSheet1.docx]

Supplementary Material

# Supplementary Tables

## Table S1: Search strategy

**Pubmed**

((((((((((((((((((((((((((((((((((((((((((((((((((Neoadjuvant Therapy[MeSH Terms]) OR (Neoadjuvant Therapies[Title/Abstract])) OR (Therapy, Neoadjuvant[Title/Abstract])) OR (Neoadjuvant Treatment[Title/Abstract])) OR (Neoadjuvant Treatments[Title/Abstract])) OR (Treatment, Neoadjuvant[Title/Abstract])) OR (Neoadjuvant Chemoradiotherapy[Title/Abstract])) OR (Chemoradiotherapy, Neoadjuvant[Title/Abstract])) OR (Neoadjuvant Chemoradiotherapies[Title/Abstract])) OR (Neoadjuvant Chemoradiation Therapy[Title/Abstract])) OR (Chemoradiation Therapy, Neoadjuvant[Title/Abstract])) OR (Neoadjuvant Chemoradiation Therapies[Title/Abstract])) OR (Therapy, Neoadjuvant Chemoradiation[Title/Abstract])) OR (Neoadjuvant Chemoradiation Treatment[Title/Abstract])) OR (Chemoradiation Treatment, Neoadjuvant[Title/Abstract])) OR (Neoadjuvant Chemoradiation Treatments[Title/Abstract])) OR (Treatment, Neoadjuvant Chemoradiation[Title/Abstract])) OR (Neoadjuvant Chemoradiation[Title/Abstract])) OR (Chemoradiation, Neoadjuvant[Title/Abstract])) OR (Neoadjuvant Chemoradiations[Title/Abstract])) OR (Neoadjuvant Radiotherapy[Title/Abstract])) OR (Neoadjuvant Radiotherapies[Title/Abstract])) OR (Radiotherapy, Neoadjuvant[Title/Abstract])) OR (Neoadjuvant Radiation Treatment[Title/Abstract])) OR (Neoadjuvant Radiation Treatments[Title/Abstract])) OR (Radiation Treatment, Neoadjuvant[Title/Abstract])) OR (Treatment, Neoadjuvant Radiation[Title/Abstract])) OR (Neoadjuvant Radiation Therapy[Title/Abstract])) OR (Neoadjuvant Radiation Therapies[Title/Abstract])) OR (Radiation Therapy, Neoadjuvant[Title/Abstract])) OR (Therapy, Neoadjuvant Radiation[Title/Abstract])) OR (Neoadjuvant Radiation[Title/Abstract])) OR (Neoadjuvant Radiations[Title/Abstract])) OR (Radiation, Neoadjuvant[Title/Abstract])) OR (Neoadjuvant Chemotherapy[Title/Abstract])) OR (Chemotherapy, Neoadjuvant[Title/Abstract])) OR (Neoadjuvant Chemotherapies[Title/Abstract])) OR (Neoadjuvant Chemotherapy Treatment[Title/Abstract])) OR (Chemotherapy Treatment, Neoadjuvant[Title/Abstract])) OR (Neoadjuvant Chemotherapy Treatments[Title/Abstract])) OR (Treatment, Neoadjuvant Chemotherapy[Title/Abstract])) OR (Neoadjuvant Systemic Therapy[Title/Abstract])) OR (Neoadjuvant Systemic Therapies[Title/Abstract])) OR (Systemic Therapy, Neoadjuvant[Title/Abstract])) OR (Therapy, Neoadjuvant Systemic[Title/Abstract])) OR (Neoadjuvant Systemic Treatment[Title/Abstract])) OR (Neoadjuvant Systemic Treatments[Title/Abstract])) OR (Systemic Treatment, Neoadjuvant[Title/Abstract])) OR (Treatment, Neoadjuvant Systemic[Title/Abstract])) AND ((((((((((((((((((((((((((((Pancreatic Neoplasms[MeSH Terms]) OR (Neoplasm, Pancreatic[Title/Abstract])) OR (Pancreatic Neoplasm[Title/Abstract])) OR (Pancreas Neoplasms[Title/Abstract])) OR (Neoplasm, Pancreas[Title/Abstract])) OR (Neoplasms, Pancreas[Title/Abstract])) OR (Pancreas Neoplasm[Title/Abstract])) OR (Neoplasms, Pancreatic[Title/Abstract])) OR (Pancreatic Carcinoma[Title/Abstract])) OR (Carcinoma, Pancreatic[Title/Abstract])) OR (Carcinomas, Pancreatic[Title/Abstract])) OR (Pancreatic Carcinomas[Title/Abstract])) OR (Pancreatic Acinar Carcinoma[Title/Abstract])) OR (Acinar Carcinoma, Pancreatic[Title/Abstract])) OR (Acinar Carcinomas, Pancreatic[Title/Abstract])) OR (Carcinoma, Pancreatic Acinar[Title/Abstract])) OR (Carcinomas, Pancreatic Acinar[Title/Abstract])) OR (Pancreatic Acinar Carcinomas[Title/Abstract])) OR (Cancer of Pancreas[Title/Abstract])) OR (Pancreas Cancers[Title/Abstract])) OR (Pancreas Cancer[Title/Abstract])) OR (Cancer, Pancreas[Title/Abstract])) OR (Cancers, Pancreas[Title/Abstract])) OR (Pancreatic Cancer[Title/Abstract])) OR (Cancer, Pancreatic[Title/Abstract])) OR (Cancers, Pancreatic[Title/Abstract])) OR (Pancreatic Cancers[Title/Abstract])) OR (Cancer of the Pancreas[Title/Abstract]))) AND ((((Chemotherapy, Adjuvant[MeSH Terms]) OR (Drug Therapy, Adjuvant[Title/Abstract])) OR (Adjuvant Chemotherapy[Title/Abstract])) OR (Adjuvant Drug Therapy[Title/Abstract]))

Filters: Publication date from 01/01/1986 to 10/09/2023

**EMBASE**

(Chemotherapy, Adjuvant or Drug Therapy, Adjuvant or Adjuvant Chemotherapy or Adjuvant Drug Therapy).af. AND (Neoadjuvant Therapy or Neoadjuvant Therapies or Therapy, Neoadjuvant or Neoadjuvant Treatment or Neoadjuvant Treatments or Treatment, Neoadjuvant or Neoadjuvant Chemoradiotherapy or Chemoradiotherapy, Neoadjuvant or Neoadjuvant Chemoradiotherapies or Neoadjuvant Chemoradiation Therapy or Chemoradiation Therapy, Neoadjuvant or Neoadjuvant Chemoradiation Therapies or Therapy, Neoadjuvant Chemoradiation or Neoadjuvant Chemoradiation Treatment or Chemoradiation Treatment, Neoadjuvant or Neoadjuvant Chemoradiation Treatments or Treatment, Neoadjuvant Chemoradiation or Neoadjuvant Chemoradiation or Chemoradiation, Neoadjuvant or Neoadjuvant Chemoradiations or Neoadjuvant Radiotherapy or Neoadjuvant Radiotherapies or Radiotherapy, Neoadjuvant or Neoadjuvant Radiation Treatment or Neoadjuvant Radiation Treatments or Radiation Treatment, Neoadjuvant or Treatment, Neoadjuvant Radiation or Neoadjuvant Radiation Therapy or Neoadjuvant Radiation Therapies or Radiation Therapy, Neoadjuvant or Therapy, Neoadjuvant Radiation or Neoadjuvant Radiation or Neoadjuvant Radiations or Radiation, Neoadjuvant or Neoadjuvant Chemotherapy or Chemotherapy, Neoadjuvant or Neoadjuvant Chemotherapies or Neoadjuvant Chemotherapy Treatment or Chemotherapy Treatment, Neoadjuvant or Neoadjuvant Chemotherapy Treatments or Treatment, Neoadjuvant Chemotherapy or Neoadjuvant Systemic Therapy or Neoadjuvant Systemic Therapies or Systemic Therapy, Neoadjuvant or Therapy, Neoadjuvant Systemic or Neoadjuvant Systemic Treatment or Neoadjuvant Systemic Treatments or Systemic Treatment, Neoadjuvant or Treatment, Neoadjuvant Systemic).af. AND (Pancreatic Neoplasms or Neoplasm, Pancreatic or Pancreatic Neoplasm or Pancreas Neoplasms or Neoplasm, Pancreas or Neoplasms, Pancreas or Pancreas Neoplasm or Neoplasms, Pancreatic or Pancreatic Carcinoma or Carcinoma, Pancreatic or Carcinomas, Pancreatic or Pancreatic Carcinomas or Pancreatic Acinar Carcinoma or Acinar Carcinoma, Pancreatic or Acinar Carcinomas, Pancreatic or Carcinoma, Pancreatic Acinar or Carcinomas, Pancreatic Acinar or Pancreatic Acinar Carcinomas or Cancer of Pancreas or Pancreas Cancers or Pancreas Cancer or Cancer, Pancreas or Cancers, Pancreas or Pancreatic Cancer or Cancer, Pancreatic or Cancers, Pancreatic or Pancreatic Cancers or Cancer of the Pancreas).af.

**Web of Science**

(TS=(Chemotherapy, Adjuvant) OR TS=(Drug Therapy, Adjuvant) OR TS=(Adjuvant Chemotherapy) OR TS=(Adjuvant Drug Therapy)) AND (TS=(Pancreatic Neoplasms) OR TS=(Neoplasm, Pancreatic) OR TS=(Pancreatic Neoplasm) OR TS=(Pancreas Neoplasms) OR TS=(Neoplasm, Pancreas) OR TS=(Neoplasms, Pancreas) OR TS=(Pancreas Neoplasm) OR TS=(Neoplasms, Pancreatic) OR TS=(Pancreatic Carcinoma) OR TS=(Carcinoma, Pancreatic) OR TS=(Carcinomas, Pancreatic) OR TS=(Pancreatic Carcinomas) OR TS=(Pancreatic Acinar Carcinoma) OR TS=(Acinar Carcinoma, Pancreatic) OR TS=(Acinar Carcinomas, Pancreatic) OR TS=(Carcinoma, Pancreatic Acinar) OR TS=(Carcinomas, Pancreatic Acinar) OR TS=(Pancreatic Acinar Carcinomas) OR TS=(Cancer of Pancreas) OR TS=(Pancreas Cancers) OR TS=(Pancreas Cancer) OR TS=(Cancer, Pancreas) OR TS=(Cancers, Pancreas) OR TS=(Pancreatic Cancer) OR TS=(Cancer, Pancreatic) OR TS=(Cancers, Pancreatic) OR TS=(Pancreatic Cancers) OR TS=(Cancer of the Pancreas)) AND (TS=(Pancreatic Neoplasms) OR TS=(Neoplasm, Pancreatic) OR TS=(Pancreatic Neoplasm) OR TS=(Pancreas Neoplasms) OR TS=(Neoplasm, Pancreas) OR TS=(Neoplasms, Pancreas) OR TS=(Pancreas Neoplasm) OR TS=(Neoplasms, Pancreatic) OR TS=(Pancreatic Carcinoma) OR TS=(Carcinoma, Pancreatic) OR TS=(Carcinomas, Pancreatic) OR TS=(Pancreatic Carcinomas) OR TS=(Pancreatic Acinar Carcinoma) OR TS=(Acinar Carcinoma, Pancreatic) OR TS=(Acinar Carcinomas, Pancreatic) OR TS=(Carcinoma, Pancreatic Acinar) OR TS=(Carcinomas, Pancreatic Acinar) OR TS=(Pancreatic Acinar Carcinomas) OR TS=(Cancer of Pancreas) OR TS=(Pancreas Cancers) OR TS=(Pancreas Cancer) OR TS=(Cancer, Pancreas) OR TS=(Cancers, Pancreas) OR TS=(Pancreatic Cancer) OR TS=(Cancer, Pancreatic) OR TS=(Cancers, Pancreatic) OR TS=(Pancreatic Cancers) OR TS=(Cancer of the Pancreas))

**Cochrane Library**

([Chemotherapy, Adjuvant] explode all trees) AND ([Neoadjuvant Therapy] explode all trees) AND ([Pancreatic Neoplasms] explode all trees)

## Table S2: Study protocol

**PICOS-elements**

population, intervention, comparison (not applicable for this study), outcome, study design

***P*:**

Patients with pancreatic cancer after neoadjuvant therapy and radical pancreatectomy

***I*:**

Receiving adjuvant chemotherapy

***O*:**

overall survival, recurrence-free survival, recurrence-free survival and tumor-specific survival

***S*:**

Cross-sectional studies, case-control studies, baseline data from cohort studies and randomized controlled trials

**Eligibility criteria**

**Type of publication**

- Cross-sectional studies, case-control studies, baseline data from cohort studies and randomized controlled trials reporting the Benefits of Adjuvant Chemotherapy in Patients with Pancreatic Cancer Undergoing Radical Pancreatectomy after Neoadjuvant Therapy
- Case reports were excluded
- Studies not reporting individual data, such as reviews, editorials, reports, prefaces, and clinical guidelines, were excluded
- Publications that were not peer-reviewed were excluded (e.g., conference abstracts and book chapters)

**Time period**

- From inception of database through September 10, 2023

**Number of cases**

- >5 cases

**Language**

- English language

## Table S3: Newcastle-Ottawa Scale (NOS)

|  | **Selection** | | | | |  | **Outcome** | | |  |
| --- | --- | --- | --- | --- | --- | --- | --- | --- | --- | --- |
| **Study (First author/year)** | | Representativeness of exposed cohort | Selection of nonexposed cohort | Ascertainment of exposure | Absence of outcome at start of study | **Comparability** | Outcome assessment | Length of follow-up | Adequacy of follow-up | Total scores |
| Olecki et al (2021) | | ***** | ***** | ***** | ***** | ***** | ***** | **-** | ***** | **7** |
| Kamarajah et al (2021) | | ***** | ***** | ***** | ***** | ****** | ***** | **-** | ***** | **8** |
| Ma et al (2019) | | ***** | ***** | ***** | ***** | ***** | ***** | ***** | ***** | **8** |
| Sugawara et al (2023) | | ***** | ***** | ***** | ***** | ****** | ***** | ***** | ***** | **9** |
| Bolm et al (2022) | | ***** | ***** | ***** | ***** | **-** | ***** | **-** | ***** | **6** |
| Zhang et al (2022) | | ***** | ***** | ***** | ***** | **-** | ***** | ***** | ***** | **7** |
| van Roessel et al (2020) | | ***** | ***** | ***** | ***** | **-** | ***** | ***** | ***** | **7** |
| Hammad et al (2023) | | ***** | ***** | ***** | ***** | **-** | ***** | **-** | ***** | **6** |
| de Geus et al (2018) | | ***** | ***** | ***** | ***** | **-** | ***** | **-** | ***** | **6** |
| Barnes et al (2017) | | ***** | ***** | ***** | ***** | **-** | ***** | ***** | ***** | **7** |
| Maggino et al (2023) | | ***** | ***** | ***** | ***** | **-** | ***** | **-** | ***** | **6** |
| Perri et al (2020) | | ***** | ***** | ***** | ***** | ****** | ***** | **-** | ***** | **8** |
| Ivey et al (2022) | | ***** | ***** | ***** | ***** | **-** | ***** | **-** | ***** | **6** |
| Pu et al (2023） | | ***** | ***** | ***** | ***** | ****** | ***** | **-** | ***** | **8** |
| Lee et al (2023) | | ***** | ***** | ***** | ***** | ****** | ***** | ***** | ***** | **9** |
| Drake et al (2020) | | ***** | ***** | ***** | ***** | ****** | ***** | ***** | ***** | **9** |

# Supplementary Figures


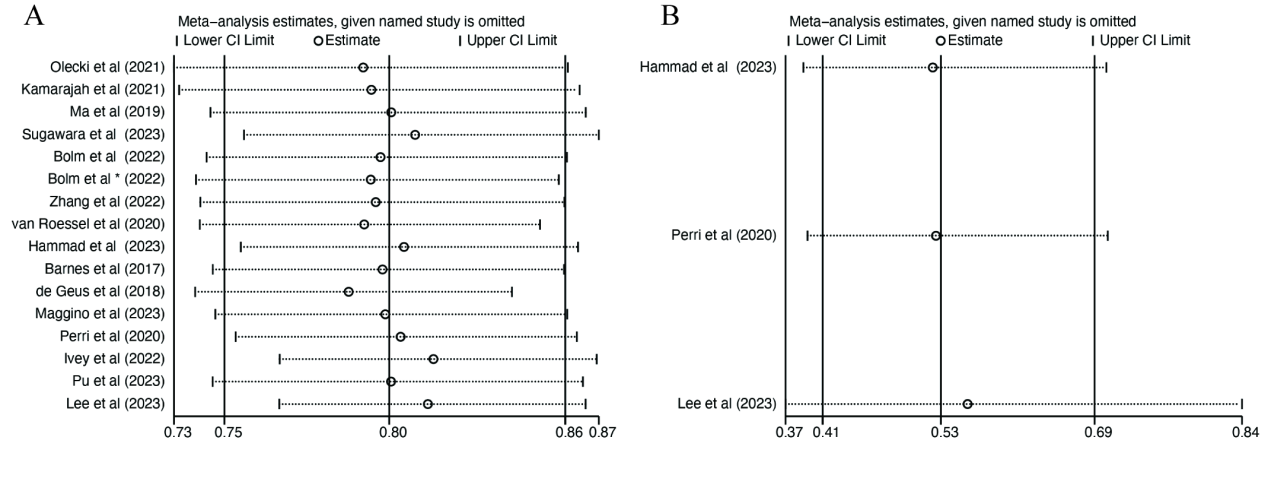


**Supplementary Figure 1.** Forest plots of 15 studies included：(A)retrospective studies, (B)prospective studies.


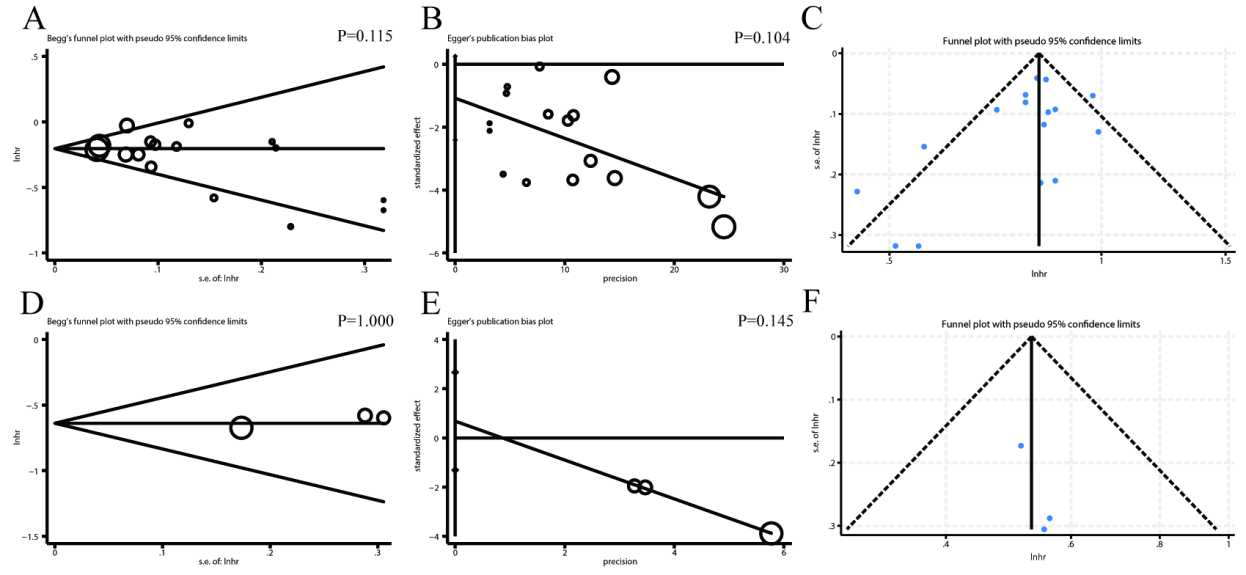


**Supplementary Figure 2.** Begg plots, Egger plots and funnel plots of OS and PFS: (A)begg plot of OS, (B)egger plot of OS, (C)funnel plot of OS, (D)begg plot of PFS, (E)egger plot of PFS, (F)funnel plot of PFS**.**


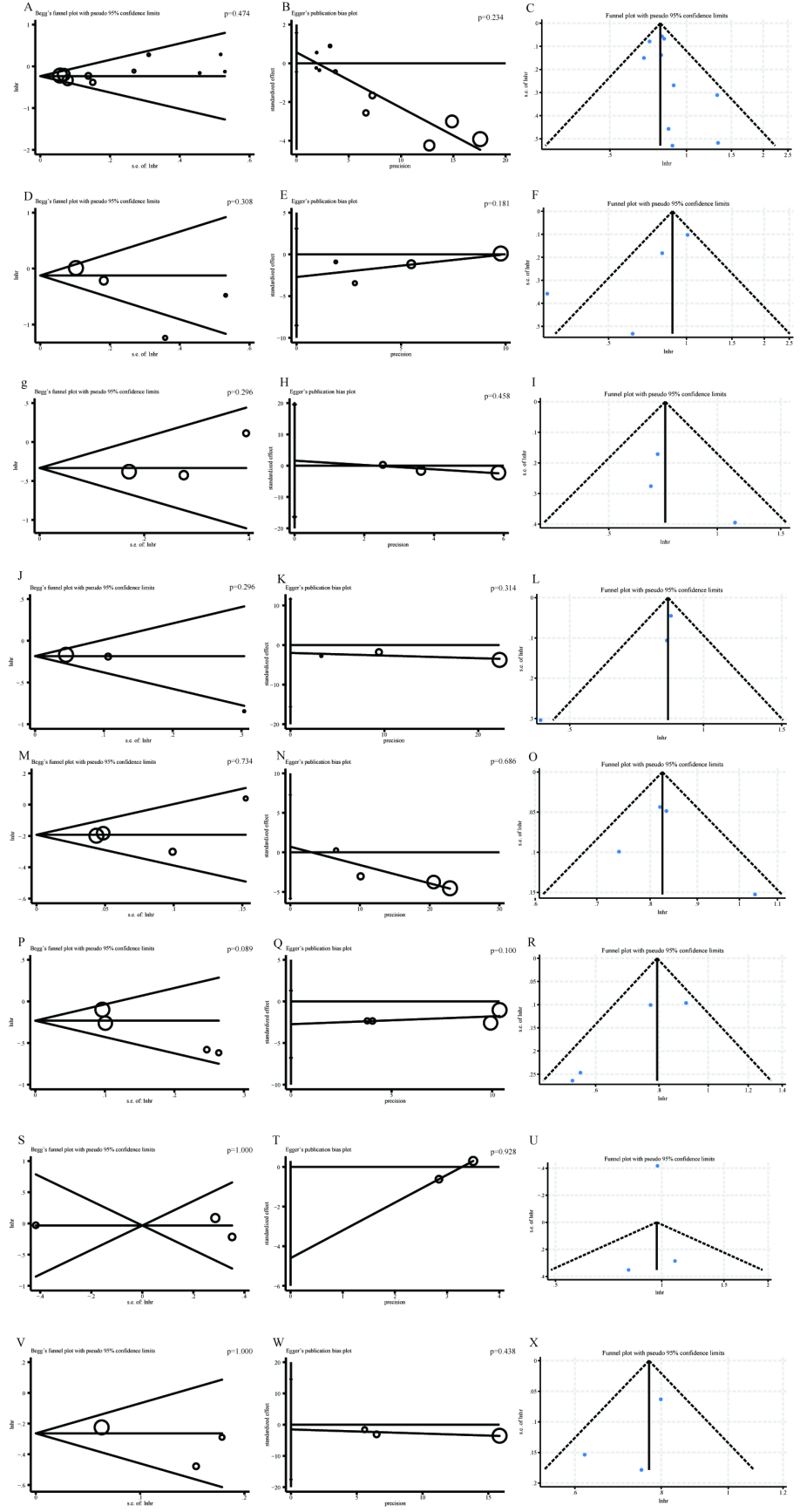


**Supplementary Figure 3.** Begg plots, egger plots and funnel plots of subgroup analysis:(A)begg plot of N0, (B)egger plot of N0, (C)funnel plot of N0, (D)begg plot of N2, (E)egger plot of N2, (F)funnel plot of N2, (G)begg plot of <2cm, (H)egger plot of <2cm, (I)funnel plot of <2cm, (J)begg plot of >2cm, (K)egger plot of >2cm, (L)funnel plot of >2cm, (M)begg plot of R0, (N)egger plot of R0, (O)funnel plot of R0, (P)begg plot of R1, (Q)egger plot of R1, (R)funnel plot of R1, (S)begg plot of high-grade group, (T)egger plot of high grade group, (U)funnel plot of high-grade group, (V)begg plot of low-grade group, (W)egger plot of low-grade group, (X)funnel plot of low-grade group.
